# A Case of Advanced Gastric Cancer with Folfiri as a Preoperative Chemotherapy

**DOI:** 10.1155/2019/1352173

**Published:** 2019-11-29

**Authors:** Hieu Van Nguyen, Hung Van Nguyen, Long Thanh Nguyen, Nga Quynh Pham, Hau Xuan Nguyen, Hien Xuan Nguyen, Vuong Thi Nguyen

**Affiliations:** ^1^Department of Oncology, Hanoi Medical University, Hanoi, Vietnam; ^2^Department of Oncology and Palliative Care, Hanoi Medical University Hospital, Hanoi, Vietnam

## Abstract

**Introduction:**

In advanced gastric cancer, preoperative chemotherapy is associated with survival benefit. FOLFIRI has demonstrated promising results in terms of survival and tolerance, especially in patients with poor performance status.

**Case Presentation:**

A 59-year-old male, diagnosed with pT4bN2M0 gastric cancer, underwent gastrointestinal anastomosis and three cycles of EOX chemotherapy. Due to disease progression, he was switched to FOLFIRI regimen. After 12 cycles, the patient received a subtotal gastrectomy and D2 lymphadenectomy. Microscopic examination achieved pCR, and the patient has been surviving 34 months without recurrence. No severe toxicities of chemotherapy were recorded.

**Conclusions:**

FOLFIRI might be a safe and effective option in neoadjuvant treatment for advanced gastric cancer among patients with poor performance status or progression after first-line chemotherapy.

## 1. Introduction

Gastric cancer currently is the third most frequent cause of cancer-related deaths worldwide [1]. In Vietnam, there were approximately 17,527 new cases and 15,065 deaths due to gastric cancer in 2018 [1].

Gastric cancer treatment is a multidisciplinary strategy, in which surgery plays an essential role. As a means of downstaging prior to an attempt of curative resection, recent guidelines has recommended preoperative chemotherapy, especially for patients with locally advanced gastric cancer [2]. Various clinical trials have been conducted to investigate the efficacy of preoperative regimens, such as ECF/ECX (epirubicin, cisplatin, and 5-FU/capecitabine) in MAGIC and REAL 2 trials and FLOT (5-FU, leucovorin, oxaliplatin, and docetaxel) in FLOT4-AIO trial [3–5]. These regimens, however, have significant toxicity and cannot be administered to patients with poor performance status. Moreover, in cases that are unresponsive to the above regimens, the second-line treatment options are currently controversial and have remarkably lower response rates than the first-line regimen [6].

FOLFIRI (5-FU, leucovorin, and irinotecan) regimen in advanced gastric cancer treatment has been evaluated in some phase III trials, revealing promising results in terms of response rate, overall survival, and tolerance [7, 8]. To our knowledge, there are limited evidences on using FOLFIRI as a preoperative chemotherapy in patients with locally advanced gastric cancer. In this article, we reported a case diagnosed as advanced gastric cancer treated with second-line preoperative FOLFIRI regimen and following gastrectomy, resulting in a pathological complete response (pCR).

## 2. Case Presentation

A 59-year-old male, with a history of hypertension and type 2 diabetes, came to a national hospital complaining of epigastric pain, early satiety, nausea, and vomiting. On esophagogastroduodenoscopy, a pyloric ulcer was found which caused gastric outlet obstruction. A biopsy was done, revealing a poorly differentiated adenocarcinoma with signet ring cell features. The patient was diagnosed as gastric carcinoma with pyloric stenosis and then underwent exploratory surgery. During operation, the tumor was found at the antrum and pylorus region invading the duodenum, the head of the pancreas and the mesentery of transverse colon and small intestine, along with multiple enlarged and hard regional lymph nodes. A gastrointestinal anastomosis and a peritoneal lesion biopsy were performed. The pathology result of peritoneal lesion was signet ring cell carcinoma (SRCC), confirming the postoperative diagnosis of advanced gastric cancer (pT4bN2M0).

Afterwards, the patient was transferred to our institution for chemotherapy. After three cycles of EOX regimen (epirubicin, oxaliplatin, and capecitabine) [2], the disease was found to have progression. An abdominal CT scan at this time showed a broadly invasive gastric tumor, multiple enlarged lymph nodes, and secondary mesenteric lesions. Laboratory data indicated an increase in the level of tumor markers compared to pretreatment values, including CEA 12.47 ng/mL (from 3.91 ng/mL) and CA19-9 9.82 U/mL (from 5.99 U/mL).

**Figure 1:**
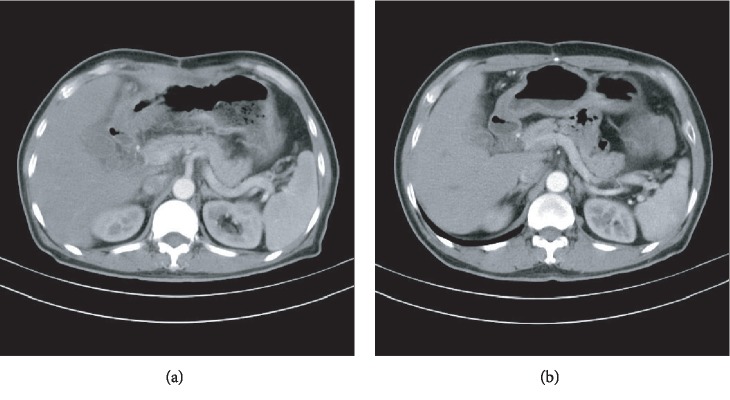
CT scan images of the patient: (a) after 3 cycles of EOX regimen and (b) after 12 cycles of FOLFIRI regimen.

We accordingly changed the chemotherapy regimen to FOLFIRI (5-fluorouracil, calcium folinate, and irinotecan) [2]. After two months (4 cycles), the tumor markers reduced below a normal range and CT scan also showed less-invasive lesions. Another CT scan done after six months (12 cycles) only showed a wall thickening of the gastric antrum (41 mm in length) with no surrounding fatty infiltration, no enlarged lymph nodes, and a well confined cyst posterior to the head of the pancreas (Figure 1). Endoscopic examination revealed no gross lesion of gastric mucosa. FOLFIRI chemotherapy has down-staged the patient from T4bN2M0 to ycT3N0M0. During chemotherapy treatment, the only significant toxicity was anemia which did not require blood transfusion and no grade 3-4 toxicities were recorded.

Then, we decided to perform a subtotal gastrectomy and a D2 lymphadenectomy on this patient. During surgery, the surgeons recorded a thickening of the antrum, without any enlarged lymph nodes or peritoneal metastases. The postoperative pathology result was gastric chronic inflammation, fibrosis, and intestinal metaplasia, with negative lymph nodes (7/7 nodes of stations 1, 3, 7, and 9 and 2/2 nodes of small intestinal mesentery) as well as negatively greater omentum lesions, giving a postop stage of ypT0N0M0. The patient was closely followed up afterwards. No adverse events were recorded during the follow-up, and a CT scan was performed 34 months after the second operation showed no recurrence.

## 3. Discussion

In this paper, we reported a patient with locally advanced gastric cancer, unresponsive to first-line chemotherapy (EOX regimen), then being treated with FOLFIRI regimen. Normally, patients who are switched to second-line regimen have poor prognosis, in which treatment is mostly palliative. However, in our case, the patient who was initially inoperable and subjected to palliative chemotherapy only became a candidate for curative resection after receiving 12 cycles of FOLFIRI. Postoperative microscopic examination demonstrated a pCR, and no recurrence has been detected 34 months after surgery.

The main purposes of neoadjuvant therapy are to downstage the tumor and increase the opportunity of curative resection. In some large-scaled clinical trials including MAGIC and REAL-2, EOX/ECF/ECX regimen has been shown to increase the rate of curative resection, improve overall survival (OS) and progression-free survival (PFS), and minimize local recurrence and distant metastases [3, 4]. With this evidence, EOX/ECF/ECX regimen had become the standard treatment at the time our patient was diagnosed. However, after three cycles of EOX, his imaging and laboratory tests indicated a disease progression. Although many second-line regimens have been evaluated, such as docetaxel-cisplatin and irinotecan-cisplatin, there has been still no consensus on which regimen is superior to others [2]. In 2014, a phase III clinical trial in France, comparing the efficacy of FOLRIFI and ECX regimen in patients with advanced gastric cancer, demonstrated no significant difference in response rate, OS, and PFS between the two regimens, but better tolerance in the FOLFIRI arm [7]. Especially, in cases that were unresponsive to first-line ECX, second-line FOLFIRI still achieved a response rate of 13.7% [7]. Until now, there have been some additional studies on chemotherapy options for advanced gastric cancer. A docetaxel-based regimen (FLOT) has been shown to have higher pathological response rate compared to EOX/ECF (16% vs. 6%), as well as better OS and PFS [9]. As a result, this regimen has been recommended to be the new standard treatment. However, its toxicity rate was considerably high, including 52% with grade 3-4 neutropenia and 12% with infection [9]. FOLFIRI regimen, meanwhile, is well-tolerated with a hematological toxicity rate of 38% [7]. Therefore, FOLFIRI could be an effective regimen for patients with poor performance status or those who are unresponsive to first-line treatment.

In this case, the patient with a SRCC, a pathological type commonly considered to have poor prognosis, achieved a pCR. pCR is an important factor in the prognosis of gastric cancer. Patients with pCR have a significantly lower relative risk (RR) of death from any cause (RR for one-year, three-year, and five-year mortalities of 0.5, 0.334, and 0.44, respectively) [10]. Many factors could be related to the pCR rate, including chemotherapy regimen, neutrophil-lymphocyte ratio, depth of tumor invasion, and histopathological type [9, 11]. Previous studies have shown that there might be a difference in molecular features, carcinogenesis, and chemosensitivity profile of SRCC compared to non-SRCC [12]. In two phase 3 clinical trials, adding irinotecan to chemotherapy regimen was associated with a longer survival time in patients with Lauren's diffuse type gastric cancer [13, 14]. Further studies are needed to clarify the reason behind this heterogeneity in the response to chemotherapy between different pathological types.

## 4. Conclusion

Selecting chemotherapy regimen depends on various factors, including patient's performance status and pathological type. In cases with poor performance status, progression after first-line chemotherapy, or signet ring cell carcinoma, FOLFIRI might be a safe and effective option.

## Additional Points

*Highlights*. In some cases, patients with advanced gastric cancer have good response to FOLFIRI regimen, even with pCR. pCR is an important prognostic factor, which is associated with a significantly longer survival time and decreased recurrence rate.
